# Predicted distribution of curl-leaf mountain mahogany (*Cercocarpus ledifolius*) in the Bighorn Canyon National Recreation Area

**DOI:** 10.1371/journal.pone.0317146

**Published:** 2025-02-03

**Authors:** Robert E. Kissell, Michael T. Tercek, David P. Thoma, Kristin L. Legg

**Affiliations:** 1 Department of Biology, Tennessee Technological University, Cookeville, TN, United States of America; 2 Walking Shadow Ecology, Gardiner, MT, United States of America; 3 National Park Service Inventory and Monitoring Program, 2327 University Way, Bozeman, MT, United States of America; Oregon State University, UNITED STATES OF AMERICA

## Abstract

Distributions of plants are expected to change in response to climate change, but the relative probability of that change is often unknown. Curl-leaf mountain mahogany (*Cercocarpus ledifolius*), an important browse species used by ungulates as forage and cover across the western US, is thought to be moderately to highly vulnerable to climate change this century, and a reduction in curl-leaf mountain mahogany occurrence may negatively impact ungulates reliant upon it. A combination of probability density estimation and vector analysis was used to predict curl-leaf mountain mahogany distribution across the species range relative to climate space and how that relationship would affect curl-leaf mountain mahogany at a local scale. Locally, we used the curl-leaf mountain mahogany population at the Bighorn Canyon National Recreation Area (BICA) in Montana and Wyoming for comparison. We modeled the probability of curl-leaf mountain mahogany occurrence across its distribution using water balance data to spatially and temporally assess the vulnerability of a population at a local scale. Modeled probabilities of occurrence and vector analysis indicated the species to remain in some areas within BICA but will be vulnerable in others given the predicted changes in temperature and precipitation in BICA if historical trajectories continue. This information allows managers to direct limited resources to other management actions by using the best available science to inform decisions. Other curl-leaf mountain mahogany populations currently inhabiting wetter, drier sites may follow a similar trajectory as the effects of climate change manifest. The approach used serves as a model to assess the predicted trend for species-specific plant communities of concern that may be adversely affected by climate change.

## Introduction

Climate change has influenced plant distribution and survival resulting in changing plant communities [1–3]. Plant species affected by climate change typically alter their distributional range either in latitude or elevation [4]. Plant populations that live in the most extreme habitats of their species’ range are more vulnerable to extirpation as climate change pushes them beyond physiological limits. Predicting the probability of occurrence of tree and shrub species in areas more vulnerable to extirpation is of management interest, especially if those plants serve as key forage species for large mammalian herbivores.

Climate change is expected to affect water balance relationships in trees and shrubs, especially in semi-arid and arid ecosystems, such that tree and shrub species are expected to suffer mortality at locations where physiological tolerances are exceeded [5]. Plant community changes have been evaluated for the continental United States, across several regionally occurring vegetation types, and within C_3_ and C_4_ grass communities [6–8]. The continental, regional and species-group findings are key to understanding present and future water balance relationships.

Temperature and precipitation trends have been used often to explain changes in plant distribution, but variables derived from a water balance model are usually more effective because they are more closely related to physical and biological processes [9–12]. For instance, plants use soil moisture rather than snow or rain directly, and not all precipitation is available to plants because some runs off or infiltrates below rooting zones. Two primary components of the water balance relationship [9] affecting latitudinal and elevation changes are actual evapotranspiration (AET) and climatic water deficit (CWD). The relationship between unmet water demand, represented by CWD, and plant growth, represented by AET, may be used to assess plant community changes [6, 13]. CWD is a good predictor of tree and shrub distributions and temporal changes in tree and shrub communities in semi-arid landscapes [14–16]. For example, increases in water deficits have been related to declines in tree densities and shifts toward more oak-dominated landscapes in the last century in California [17]. Because of the mechanistic relationship that AET and CWD have with plant communities, water balance relationships are used to predict plant community presence and diversity at global or continental scales [18, 19], plant species at local or regional scales [14], and animal species distributions across spatial scales [20].

**Fig 1:**
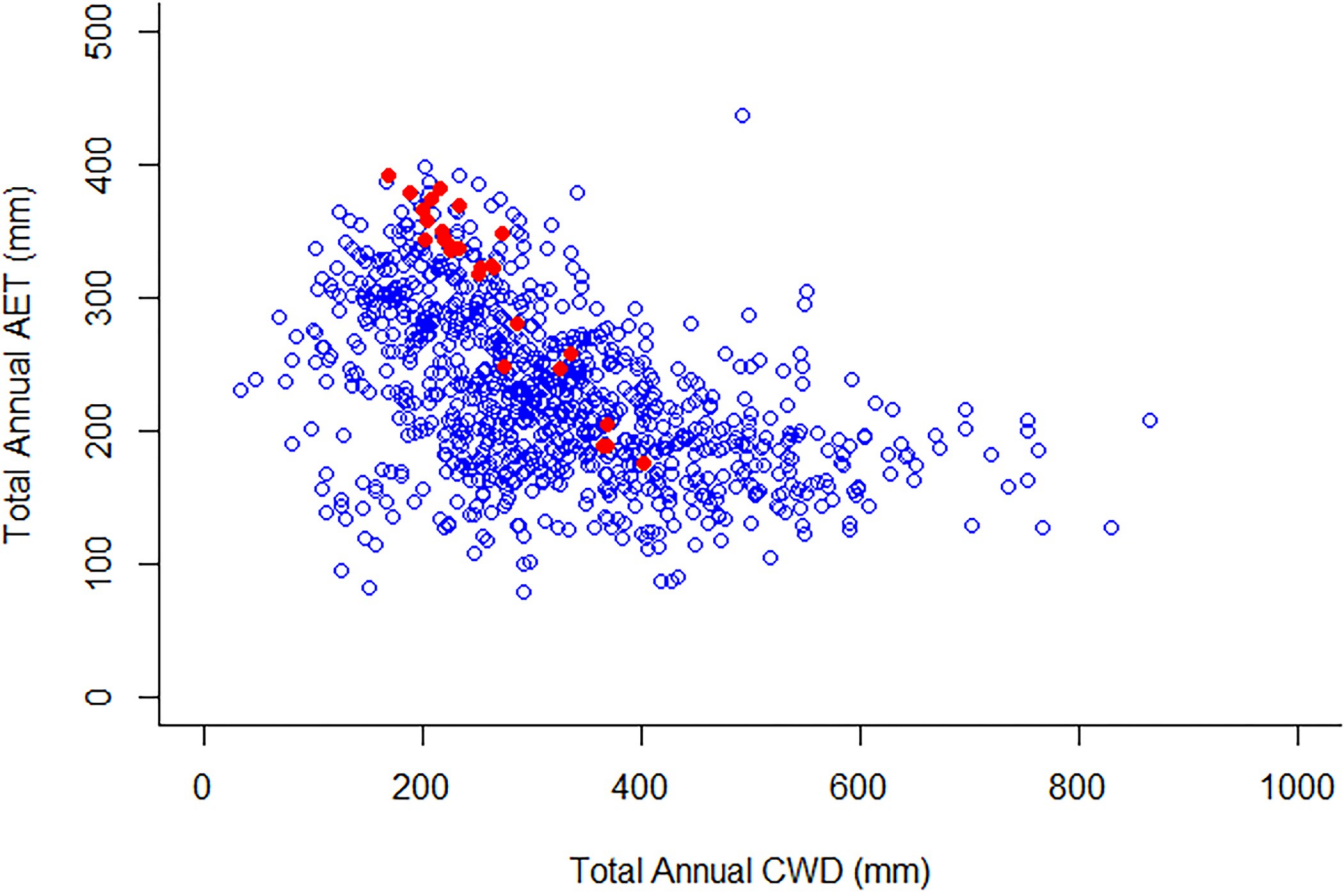
Relationship between Actual Evapotranspiration (AET) and Climatic Water Deficit (CWD) for curl-leaf mountain mahogany (*Cercocarpus ledifolius*) for known curl-leaf mountain mahogany locations using 1 km^2^ grid cells throughout the continental United States obtained from the Global Biodiversity Information Facility (gbif.org; open blue circles) and locations using 1 km^2^ grid cells in the Bighorn Canyon National Recreation Area (solid red circles).

Curl-leaf mountain mahogany (*Cercocarpus ledifolius*; hereafter, mountain mahogany) is found across the Intermountain West as a climax community species [21–23], as a co-dominant with Utah juniper (*Juniperus osteosperma*) and other species [24, 25], or as a seral species [26]. The distribution is scattered and disjunct spatially due to physiognomy and physiology [27], and the species occurs in stands ranging in size from several square meters to thousands of hectares [26]. Mountain mahogany is a long-lived (i.e., > 100 yrs) tree species [28], sometimes occurring in shrub form under conditions of limited precipitation and infertile soils, and it is often found in small stands isolated from fire [26, 29, 30]. Mountain mahogany is most often found on warm, dry, rocky ridges and slopes at high elevations on mostly southern exposures. Mountain mahogany approaches or exceeds elevations of 3,000 m in the southern parts of its range and as low as 1,000 m in some areas of the northern portions of its range, but most commonly it grows between 600 m and 3,000 m on both sides of the continental divide [31, 32]. Mountain mahogany has the capacity to fix nitrogen via nitrogen-fixing nodules; this helps explain how the species can successfully occupy the infertile sites with which it is so regularly associated [33–35]. Mountain mahogany is a drought-resistant species [26] but is considered moderately to highly vulnerable to climate change this century [36, 37]. The AET-CWD climate space based on 970 known locations of mountain mahogany within the continental United States (Fig 1) demonstrates the breadth of climate-related conditions under which the species may be present. Sampling locations in Bighorn Canyon National Recreation Area (BICA) used in this study occurred across that AET-CWD climate space range.

Mountain mahogany occurs in BICA in separate, isolated stands as well as mixed stands with Utah juniper. Bighorn sheep (*Ovis canadensis*) inhabit BICA along with mule deer (*Odocoileus hemionus*) and horses (*Equus caballus*). Given bighorn sheep rely heavily (i.e., up to 68%) on mountain mahogany throughout the year as a key forage species [38], a reduction or loss of mountain mahogany is expected to have negative effects on the bighorn sheep population in BICA.

To provide management or mitigation insight for populations expected to be vulnerable to a changing climate, it is key to understand how species-specific relationships at the local scale relate to the regional or continental scale. We provide a method to estimate the probability of occurrence based on current climate space data from the regional scale to predict the climate space of mountain mahogany at the local scale using water balance variables to assess the distributional response to a changing climate. As a result, this project was developed to increase our understanding of mountain mahogany vulnerability to future climates.

## Material and methods

### Study area

The Bighorn Canyon National Recreation Area is in Bighorn County, Wyoming, and Bighorn and Carbon counties, Montana. It encompasses approximately 48,500 ha with elevations from 975 m to 2,134 m. A precipitation gradient exists with colder, drier conditions in the south compared to the north. Mean maximum and mean minimum annual temperatures in the south are 14.6°C and -0.3°C, respectively; mean maximum and mean minimum annual temperatures in the north are 16.2°C and 2.7°C, respectively. Mean annual precipitation also increases latitudinally with the mean annual precipitation from 16.9 cm in the south to 45.3 cm in the north [39].

Soils are composed of sandstone, limestone, shale, dolomite, and alluvial deposits [40, 41]. Soil depth varies but has been described as severely depleted (i.e., soil depth 0–25.4 cm) [42]. Mountain mahogany occurs on very shallow soils between 1,100 and 1,550 m elevation. Slope averages 10.7% (12.9% SD), ranging from flat to 82.2%. Aspects facing north (315° - 45°) and south (135° - 225°) account for approximately 29.4% and 21.7% of the area, respectively. Komp et al. [39] described four vegetative communities based on nine vegetative types described by Knight et al. [43]. The Juniper, Pine, Mountain Mahogany Community is the largest of the four communities, representing approximately 40% of the area [43, 44].

### Modeling data

We modeled the probability of occurrence of the climate space of mountain mahogany using the kernel density estimation technique [45, 46]. Presence on BICA (n = 78) was based on data collected from randomly selected 30 X 30 m plots from a 30 X 30 m sampling space of plots covering pure stands of mountain mahogany and mixed mountain mahogany/Utah juniper stands [47]. Locations for plot centers were used for BICA locations (S1 Table). The National Park Service, Bighorn Canyon National Recreation Area approved field access (permit number BICA-2017-SCI-0004). Range-wide location data (S2 Table) for mountain mahogany were obtained from the Global Biodiversity Information Facility [48]. The GBIF is an open-source, international outlet for occurrence, checklists, and sampling-event datasets. Datasets for mountain mahogany in the GBIF were provided in descending sample size by iNaturalist, University of Nevada Herbarium, UCR-University of California, Riverside Herbarium—Vascular Plants, BLM-National Landscape Monitoring Framework-Plants, The New York Botanical Garden Herbarium, Arizona State University Vascular Plant Herbarium, Intermountain Herbarium (Vascular Plants and Algae), RSA-California Botanic Garden Herbarium, Deaver Herbarium (Northern Arizona University), Baylor University Herbarium, and 88 other repositories (S3 Table). The GBIF recorded 6,336 observations of mountain mahogany across its range. Field observations represented 46% of locations and approximately 54% of locations were based on preserved specimens. We filtered observations that had no location, locations that had > 0.5 km of uncertainty, and locations within municipalities. Additionally, we filtered multiple locations within each of the 1 km^2^ AET and CWD grid cells to prevent psuedoreplication, which resulted in a total of 970 locations (S2 Table). All locations were recorded in latitude and longitude and represented the distributional range of the species.

Plant communities are often characterized by their location on a bivariate AET vs CWD plot representing a climate space [6, 9, 11], as AET and CWD are more closely related to physical and biological processes than temperature and precipitation [10–12]. We used the daily single bucket Thornthwaite-type one dimensional model [10, 12, 14] and daily values were aggregated to annual values prior to analysis. In the water balance model, annual AET (mm) was the amount of water that was used by plants for transpiration and annual CWD (mm) estimated the amount of unmet water needs. Additional details on methods are described in a previous study by the current authors [6] and data used here are derived from that earlier work. Total annual AET and CWD were calculated for each 1 km^2^ in North America [6] and data for annual AET and CWD were extracted for each corresponding 1 km^2^ location [49, 50].

### Modeling analyses

Fixed kernel estimation [45, 46] was performed with the kernelUD function using the adehabitatHR package in R software (ver. 2022.12.0). The Utilization Distribution is a nonparametric function giving the probability density that an object of interest is found at a point based on its spatial coordinates. A minimum of 50 locations is recommended to minimize bias and variance [46]. The probability distribution was constructed using a smoothing parameter, h, set to “href” and a bivariate normal kernel. CWD was on the x-axis, AET was on the y-axis, and probability was represented by the pixel color of the distribution of the climate space relative to the occurrence of mountain mahogany.

We used the representative concentration pathway 8.5 (RCP8.5) [51]. RCP8.5 was chosen because it represented a likely extreme scenario [51]. BICA is likely to have hot, dry conditions in the late century. Greenhouse gas emissions continue to increase and, as a result, the higher RCP8.5 projected temperature increase may be expected rather than lower increase forecast by the RCP4.5 scenarios for end of the century [52]. Annual totals of AET and CWD for each pixel in BICA were averaged across years using the GFDL-ESM2G, IPSL-CM5A-MR, MIROC-ESM-CHEM, and MRI-CGCM3 global climate models (GCM) to predict future (2070–2099) climate space relationships for mountain mahogany. These models were chosen to represent possible future temperature-precipitation scenarios [53]. The starting point for each vector was taken from 1981–2010 averages of Gridmet data for each location [52]. The ending point for each vector was taken from the 2070–2099 averages of the GCMs. Starting and ending points of vectors in two-dimensional space were defined with CWD on the horizontal axis and AET on the vertical axis. Intensity of the combined change in AET and CWD (length of the vector) was calculated as the Euclidean distance between the two points defined by the two time periods. These “change vectors” were calculated for every 1 km^2^ grid cell in BICA and provided a measure of the change in intensity and direction of the climate space at a local scale [50].

## Results

**Fig 2:**
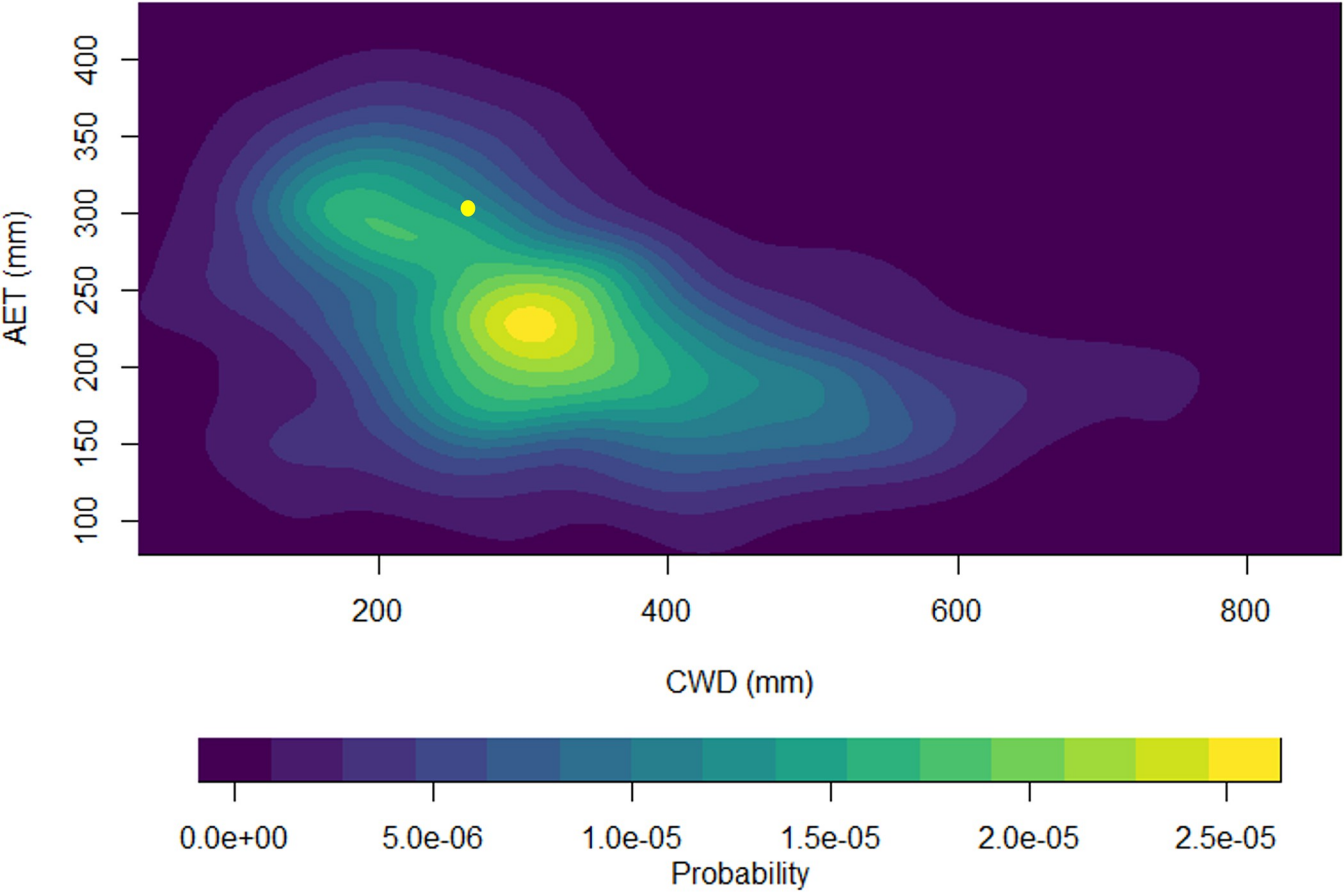
Probability distribution of annual climatic water deficit (CWD; mm/yr) vs actual evapotranspiration (AET; mm/yr) for curl-leaf mountain mahogany across its range in relation to the average AET-CWD climate space location (yellow dot) of curl-leaf mountain mahogany in the Bighorn Canyon National Recreation Area.

Probability density based on the current range-wide AET and CWD estimates (Fig 2) indicated a distribution with the maximum probability of mountain mahogany occurring where AET and CWD were approximately 228 mm and 319 mm, respectively. The probability of occurrence declined moving away from the maximum. The average AET and CWD coordinate for BICA was found close to the smallest mode (Fig 2), with AET ≈ 262 and CWD ≈ 310. The probability distribution illustrated a steep decline in the probability of occurrence relative to increasing CWD.

**Fig 3:**
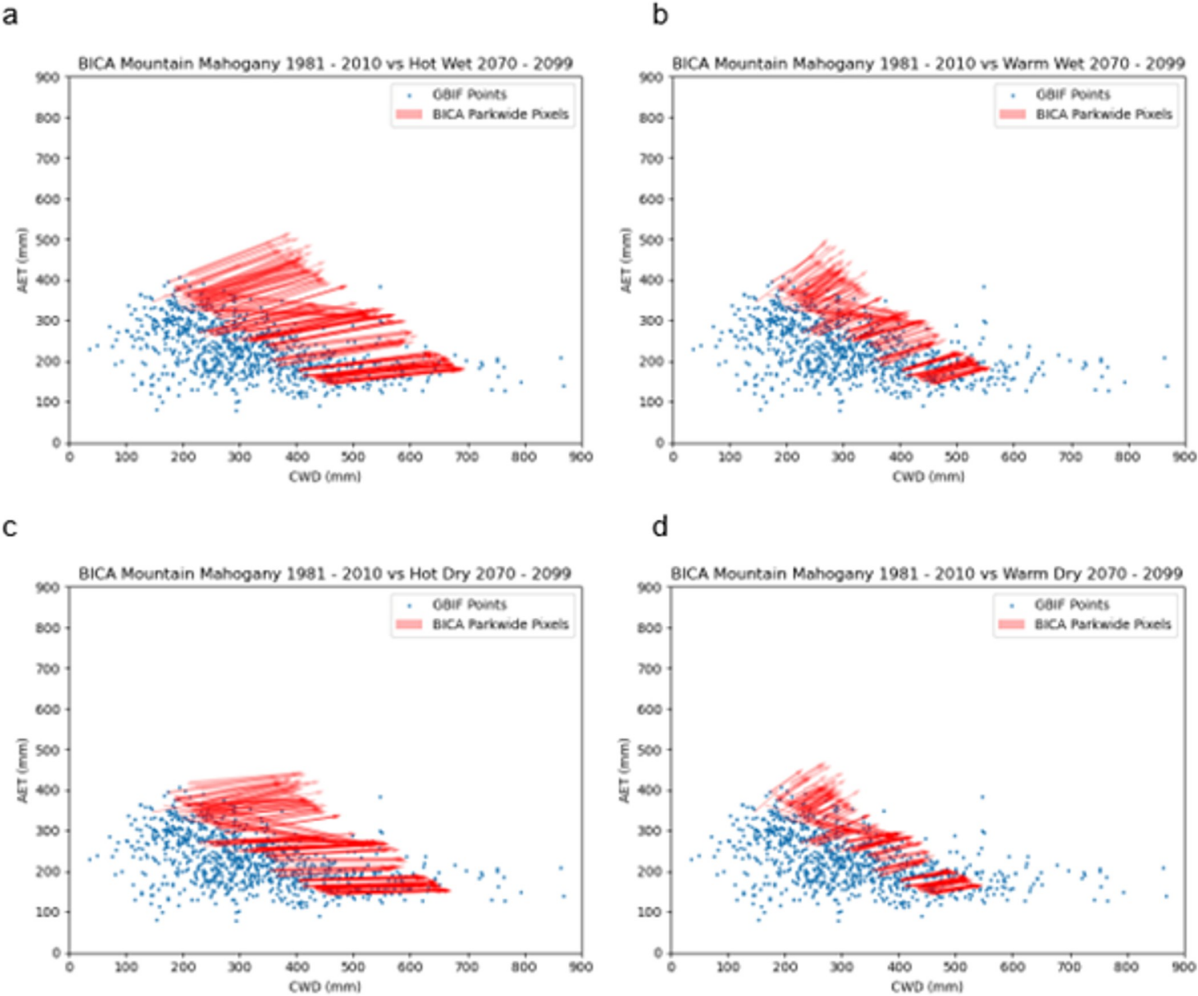
Estimated trajectory of curl-leaf mountain mahogany (arrows) in the Bighorn Canyon National Recreation Area between current (1981–2010) and late century (2070–2099) time periods relative to the AET vs CWD climate space for hot, wet conditions (a), warm, wet conditions (b), hot, dry conditions (c), and warm, dry conditions (d).

RCP8.5 scenario estimates were mixed. All models predicted an increase in temperature, but changes in precipitation varied (Table 1). Average increase in temperature was 9.9°C with warm temperatures defined as an increase ≤ 9.9°C and hot temperatures defined as an increase > 9.9°C. Average percent change in precipitation was -2.25%. Dry climate was defined as a change of ≤ -2.25% of annual precipitation and wet climate increased > -2.25%. Four future categories based on these temperature and precipitation delineations resulted in warm-wet conditions, hot-wet conditions, warm-dry conditions, and hot-dry conditions represented by the GFDL-ESM2G.rcp85, MIROC-ESM-CHEM.rcp85, MRI-CGCM3.rcp85, and IPSL-CM5A-MR.rcp85 models, respectively. Hot-wet and hot-dry vectors (Fig 3A–3C) increased more in magnitude than warm-wet and warm-dry vectors (Fig 3B–3D).

**Table 1:** Changes in temperature (°C) and precipitation (%) for selected Global Climate Models (GCM) between the periods of 1981–2010 and 2070–2099 for the Bighorn Canyon National Recreation Area.

GCM	ΔTemperature (°C)	ΔPrecipitation (%)	Categories
GFDL-ESM2G.rcp85	7.9	0.00%	Warm-Wet
MIROC-ESM-CHEM.rcp85	13.9	2.00%	Hot-Wet
MRI-CGCM3.rcp85	6.2	-5.00%	Warm-Dry
IPSL-CM5A-MR.rcp85	11.6	-6.00%	Hot-Dry

More than half of the 1 km^2^ pixels in BICA plotted outside the current bivariate climate space in the late century time period for each of the four models (Fig 3). During the late century time period, most 1 km^2^ pixels in BICA were expected to increase in AET and all 1 km^2^ pixels were expected to increase in CWD. Those 1 km^2^ pixels that decreased in AET remained in the periphery of the AET-CWD climate space. The 1 km^2^ pixels with AET values < approximately 300 mm were projected to persist within the AET-CWD climate space regardless of the temperature-precipitation combination.

## Discussion

### Probability expectations

The probability distribution (Fig 2) provided an estimate of likelihood of occurrence of mountain mahogany with respect to values of CWD and AET climate space. CWD has been identified as a prominent driver of species occurrence [12, 14, 16]. As CWD increases, the probability of occurrence decreases quickly if AET increases or remains relatively constant for much of the climate space. Probability of occurrence would likely remain constant or increase if AET decreased in those areas with AET values > 300 mm and is expected to approach zero as CWD increases.

### Change vectors

The probability distribution relationship and vector analysis both indicated that as CWD is expected to increase, AET would need to decrease to remain in the climate space in BICA. The same relationship occurred in general across the western U.S. and the trend was attributed to increasing temperature [6]. Two general trends were observed with change vectors. With few exceptions in each model, change vectors increased in AET and CWD values. The projected change is different from what is needed to keep the pixels in the climate space. Increases in CWD associated with increases in AET indicate increasing evaporative demand [16]. Even though the warm-wet and hot-wet change vectors (Fig 3A and 3B) were associated with stable or increasing percent precipitation, predicted increased temperatures would be such that AET would be expected to increase to compensate for the increase in CWD caused by the increased temperatures. Those few change vectors in each of the models that decreased in AET and increased in CWD (i.e., down and right vectors) indicate decreasing water availability [16]. Both situations were sufficient to predict future climate space in many 1 km^2^ areas in BICA beyond the current climate space observed across the species distribution.

A decrease in AET associated with an increase in CWD for selected species at the local scale has been observed in many dry vegetation communities across the western US [6–8]. CWD in BICA is expected to be similar to other dry vegetation communities across the western US [6–8], but models indicated increasing (Fig 3A, 3B–3D) or stable AET values (Fig 3C) in most of BICA which deviates from other western US dry vegetation communities. However, some other national parks, such as Sequoia National Park, exhibited similar changes from 1980–1999 to 2000–2019 [6], but those represented northwestern forested mountains or mediterranean California ecoregions.

### Scale

The AET-CWD trajectories observed at the regional scale [6, 14] was the same as at the local scale (e.g., BICA; see Fig 1) in that as CWD increased, AET decreased, and BICA locations of known mountain mahogany occurred within the greater distribution. This represents common processes occurring across scales including similar habitats and similar physiological responses for species not at the edge of their distribution (i.e., not physiologically stressed to the point of extirpation). Several dominant species across the western US in addition to mountain mahogany, such as *Prushia tridentada* and *Rhus trilobata*, have been recognized to have similar habitat requirements [38]. A hotter, drier climate should provide the same effect on other dominant species requiring similar habitat as mountain mahogany [45] and result in a similar trajectory that includes higher CWD and higher AET values. However, if climate causes AET and CWD to increase concurrently as in our work, some areas will lose mountain mahogany while other areas will still support the species.

Much of the previous research [6, 8, 9, 54] focused on a finer temporal scale of AET because of the ecophysiological relationship it has with species or community distribution [9]. AET was expected to have a greater effect in spring and early summer, and CWD was expected to have a greater effect in mid- and late summer. Our measurements were at an annual time scale that may have minimized the importance of variables influenced by seasonality. Distribution models for the Great Basin using CWD found mountain mahogany performed better than 17 other species [12]. The spring water supply was the most important water deficit predictor for this species. Plants that exist on low fertility sites such as serpentine soils, such as mountain mahogany, are expected to be more stress tolerant and less affected by climate change [55]. Mountain mahogany has fine roots in shallow soils that progressively become larger with soil depth and are often found in bedrock [29, 56]. Species with deeper roots may have a competitive advantage for soil moisture. Shallow roots of mountain mahogany are expected to take advantage of spring moisture while deeper roots may use deeper water reserves at other times of the year. Even though our analysis was conducted at a larger scale than AET and CWD operate, the spatial and temporal variation in AET and CWD on a smaller scale provides for the existence of mountain mahogany that was used to predict occurrence.

In mixed stands of mountain mahogany and Utah juniper, Utah juniper may provide shade that reduces temperatures, thereby affecting water balance on a microsite level. Increased shading may decrease evapotranspiration thereby allowing for greater soil moisture [57, 58]. The relationship between CWD and shrub species distribution is common and appears to predict distributions well [12, 59].

### Ecophysiological relationships and drought

All species live within ecological and physiological tolerances [60, 61]. AET-CWD climate space derived from the regional scale defines the physiological tolerance limits. Given the general trend for AET and CWD is to increase in the late century across most all 1 km^2^ pixels in BICA, it appears that mountain mahogany will be beyond the species physiological tolerance limits of the climate space exhibited at the regional scale for some areas in BICA. Increasing unmet water demand, represented by increasing CWD [6, 13], is expected as BICA becomes hotter and drier. Mountain mahogany requires little precipitation (250–610 mm) per year for good growth [26], but hotter and drier conditions are likely to reduce the water available for physiological processes. As more of BICA becomes hotter and drier, less habitat may be available where mountain mahogany can perform sufficiently. Mechanisms allowing for an increase in local occurrence of mountain mahogany are likely related to lower leaf surface to mass ratio and the effect of shading. Other dominant species like mountain mahogany that can withstand more severe droughts likely increase their water use efficiency at the cost of a low growth rate [62, 63] and are expected to have special structures that improve hydraulic efficiency and cavitation resistance [5, 64, 65]. Should these assumptions be met, an expectation of similar processes across multiple spatial scales may be realized.

The change in probability of mountain mahogany occurring across BICA in the future will require mountain mahogany to be able to withstand more severe droughts, as indicated by higher CWD values [7]. Species better able to withstand drought increase their water use efficiency at the cost of a low growth rate [62, 63]. The trade-off between plant carbon gain and water loss occurs through the opening of stomata, which creates strong selective pressure for drought tolerant plants [63]. Mountain mahogany, thought to be a drought-tolerant species [26], has special structures that improve hydraulic efficiency and cavitation resistance [64, 65]. Understanding functional traits, such as hydraulic efficiency and cavitation resistance, and climate metrics are crucial for developing more accurate models to make predictions about climate-driven shifts in the likelihood of distribution [4].

Severity and length of droughts may greatly affect plant community distribution. Short-term droughts have been shown to be detrimental to juniper establishment in grasslands, but long-term droughts reduced grass competition and may enhance juniper invasion following drought [61]. Should mountain mahogany have a higher stress tolerance than Utah juniper and Utah juniper experience drought sufficient to cause mortality, a transition to mountain mahogany would be expected if the water balance relationship was favorable. Arid and semi-arid climates, such as BICA experiences, are expected to have increased drought effects associated with increasing CWD values, but we did not examine the effects of drought severity or drought length on future climate space relationships. There is a paucity of information concerning the comparative effects of drought on sympatric mountain mahogany and Utah juniper stands.

### Uncertainties

We used AET without partitioning the value into evaporation and transpiration. We are just showing changes in boundary conditions for the two variables (evaporation and transpiration) combined without trying to partition them. Vegetation composition (i.e., root density and depth) and growth form [66] would change the ratio of evaporation and transpiration, which is not something we could model. We modeled the drivers of change, not the actual water balance conditions as they unfold over time as vegetation transitions occur which could alter the ratio of evaporation and transpiration.

We used annual AET and CWD values instead of seasonal values to model future AET and CWD conditions for mountain mahogany. We acknowledge seasonal AET and CWD values may capture important extremes and that annual values reduce variation. However, we modeled spatially at the regional level and temporally at the annual level to provide better matched scales. Vector change models from the regional scale applied to local modeling, thereby limiting the spatial component to BICA, emphasized the future conditions of the AET-CWD climate space on BICA.

## Conclusions

We provided a climate space model for mountain mahogany based on probability of occurrence using water balance tolerances from across the species range. Application of this model, given local water balance (i.e., AET-CWD) relationships, allows for predictions at a local scale for this species under future conditions. Individual plants and populations are impacted locally and how local habitat, specifically the AET-CWD climate space, changes will dictate occurrence. This same approach may be used to provide a climate space model for other potentially at-risk plant species.

Mountain mahogany, as a long-lived plant species occupying arid and semi-arid landscapes, has a life history that is adapted to environmental extremes but is reliant on reproductive events facilitated by above average precipitation and habitat located in protected areas to maintain recruitment. It is possible climate change will affect the frequency of these above average precipitation events impacting recruitment. Given seedling survival is affected by drought [26], sites remaining within the physiological limits for adult plants but experiencing recruitment failure due to drought is expected to lead to eventual extirpation of the species. Long-term averages do not capture this important aspect of climate variability (i.e., regeneration niche), making predictions of future habitat viability difficult.

Mountain mahogany, like some species [67–69], is expected to respond positively to projected warmer, drier environments in some areas due to climate change and become extirpated in others, all other factors being equal. Presumably, microsites at range edges are common due to fine-scale variation on slope, aspect, soil properties and drainage patterns. So, as the regional BICA climate space becomes unfavorable in the future for mahogany, it is likely that microsites will be the only places where mahogany can persist in BICA. While the probability of occurrence of mountain mahogany will decline, along with its abundance and area occupied, it may persist in microsites that remain within the climate space within which mountain mahogany is adapted. Finer scale modeling of AET, CWD, and associated topoedaphic characteristics is needed for better understanding the microsite AET-CWD climate space. Management focused on the persistence in microsites, and perhaps planting in favorable microsites, is recommended.

Many parks in the western United States have arid ecosystems and where unmet water demand occurs some species, such as curl-leaf mountain mahogany, may experience less plant growth [6, 13] due to a shift to a hotter, drier environment. Knowing the probability of species occurrence improves a land managers ability to make effective decisions and take action at the appropriate locations and time to conserve species [70]. In our study, the probability of mountain mahogany occurrence indicated mountain mahogany at BICA is likely to be vulnerable to a hotter, drier climate in the coming decades. This information allows managers to direct limited resources (e.g., time and money) to appropriate management actions, such as invasive plant removal, by using the best available science to inform decisions.

Climate is not the only force acting on mountain mahogany, however, and other potential factors (e.g., over-browsing by mammals or insects, wildland fire) or a combination thereof may affect mountain mahogany [71–73]. We recognize probability models, such as the one we used, are very effective in predicting current distributions but that other environmental variables (e.g., wildland fire) and distribution-limiting processes (e.g., over-browsing) may change over time [74]. Because of an expected hotter, drier climate and other potential factors that may affect mountain mahogany distribution which are dynamic processes that change over time, continued long-term monitoring is warranted to track the health and condition of mountain mahogany at this site and others across its distribution.

## Supporting information

S1 Table Curl-leaf mountain mahogany presence locations in each 1 km^2^ grid cell in the Bighorn Canyon National Recreation Area. (CSV)

S2 Table Curl-leaf mountain mahogany presence locations in each 1 km^2^ grid cell across the species’ distribution. (CSV)

S3 Table Sources of curl-leaf mountain mahogany location data provided by the Global Biodiversity Information Facility. (CSV)
